# Drug shortage management: A qualitative assessment of a collaborative approach

**DOI:** 10.1371/journal.pone.0243870

**Published:** 2021-04-23

**Authors:** Emily Chen, Susan Goold, Sam Harrison, Iman Ali, Ibtihal Makki, Stanley S. Kent, Andrew G. Shuman

**Affiliations:** 1 Department of Pharmacy, Michigan Medicine, Ann Arbor, Michigan, United States of America; 2 University of Michigan Medical School Center for Bioethics and Social Sciences in Medicine, Ann Arbor, Michigan, United States of America; 3 University of Michigan Institute for Healthcare Policy and Innovation, Ann Arbor, Michigan, United States of America; College of Pharmacy & Health Sciences, UNITED STATES

## Abstract

Drug shortages frequently and persistently affect healthcare institutions, posing formidable financial, logistical, and ethical challenges. Despite plentiful evidence characterizing the impact of drug shortages, there is a remarkable dearth of data describing current shortage management practices. Hospitals within the same state or region may not only take different approaches to shortages but may be unaware of shortages proximate facilities are facing. Our goal is to explore how hospitals in Michigan handle drug shortages to assess potential need for comprehensive drug shortage management resources. We conducted semi-structured interviews with diverse stakeholders throughout the state to describe experiences managing drug shortages, approaches to recent shortages, openness to inter-institutional engagement, ideas for a shared resource, and potential obstacles to implementation. To solicit additional feedback on ideas for a shared resource gathered from the interviews, we held focus groups with pharmacists, physicians, ethicists, and community representatives. Among participants representing a heterogeneous sample of institutions, three themes were consistent: (1) numerous drug shortage strategies occurring simultaneously; (2) inadequate resources and lead time to proactively manage shortages; and (3) interest in, but varied attitudes toward, a collaborative approach. These data provide insight to help develop and test a shared drug shortage management resource for enhancing fair allocation of scarce drugs. A shared resource may help institutions adopt accepted best practices and more efficiently access or share finite resources in times of shortage.

## Introduction

The World Health Organization (WHO) characterizes drug shortages as a critical global health problem [1, 2]. Drug shortages are caused by factors such as raw material shortages, regulatory influences, manufacturing difficulties, and financial implications [3, 4]. To date, the American Society of Health System Pharmacists (ASHP) has reported hundreds of active shortages each year [5] Underlying economic, logistical, political, and regulatory causes have been well described and were recently summarized in a Food and Drug Administration (FDA) report [6, 7]. The COVID-19 pandemic has only exacerbated these problems related to both supply-side challenges as well as increased demand for medications used for critically ill patients [8].

Drug shortages frequently and persistently affect healthcare institutions, providers, and patients–posing formidable financial, logistical, and ethical challenges [9]. An estimated $416 million dollars are associated with the annual labor cost of managing drug shortages in the United States [10]. At least an additional $215 million are spent purchasing alternative medications annually not to mention the substantial costs incurred when institutions seek distribution from the pharmaceutical gray market as well [4, 11]. Drug shortages can also lead to increased risk of medication errors and adverse patient outcomes [12, 13]. Based on experience at our institution, these authors believe the financial and patient impact is likely much greater.

The ASHP Guidelines on Managing Drug Product Shortages provide a thorough overview of strategic management of shortages for institutions [14]. Despite this, institutions rarely have the resources (time, information, and personnel) to follow the recommended pathways due to varying organizational cultures, decision-making processes, electronic health records, formularies, acuity levels, and inventory management practices. Due to the acute and dynamic nature of shortages, decisions may be made rapidly with only a few persons involved. Thus, little is known about how institutions and medical personnel make decisions regarding which patients should receive limited supplies of drugs or devices. In a national survey of pediatric hematologists/oncologists, nearly 65% of practitioners said they had patients directly affected by drug shortages. However, one-third did not know of a program or policy for handling drug shortages at their institution, and one-fourth did not know who makes decisions about drug allocation [15]. This illustrates a potential lack of transparency even within institutions, let alone across them.

In a 2019 national survey of health-system pharmacists, respondents indicated managing at least 50 shortages in the last year [16]. One-third of respondents reported having a dedicated shortage pharmacist and/or standing shortage committee. Responses revealed variation and uncertainty in mitigation and allocation strategies, which included hoarding available supply, requesting supply from local hospitals, transferring patients and, for many, inadequate capacity to manage concurrent drug shortages.

Given the frequency and often urgency of drug shortages, and inadequate capacity for managing them, institutional responses are likely to be conducted in isolation, uncoordinated with other institutions, and management may differ from place to place. Hospitals within the same state or region may not only take different approaches to certain shortages, but can be unaware of shortages their neighbors are facing [2]. Such variation has the potential to exacerbate disparities in access to scarce drugs, since patients and families with sufficient resources may, faced with a shortage at one institution, pursue an alternate source at another [17].

Numerous surveys, roundtables, and working groups have been convened to discuss strategies for mitigation and management of drug shortages [18–21]. A common thread of such discussion focuses on an interest in a resource to share information about drugs in short supply. One working group (specifically discussing oncology agents) suggested the development of an “accurate, comprehensive, controlled-access clearinghouse that is centrally managed and made available to health institutions and systems for sharing drug information (including expected duration, available alternatives, and sources, and contacts)” [10]. Yet we know little about how collaboration and communication could be effectively employed for a collective response to drug shortages.

Our primary goal is to explore how hospitals in Michigan handle drug shortages to assess potential need for comprehensive drug shortage management resources. Utilizing the results from the qualitative interviews, we hope to ultimately develop a shared, collaborative resource that can allow scarce drugs to be optimally utilized, patients to be treated fairly, and decision makers supported and trusted to make morally, intellectually, and emotionally complex drug shortage decisions. To ensure that such a resource would be used and valued, we explored how stakeholders would want to design, use, and implement a resource in which supply, demand, and institutional approaches are shared openly, as well as exploring the facilitators and obstacles to its use.

## Materials and methods

Qualitative data collection and analysis aims to discover “how” and “why” phenomena occur, and describe the context and explanations in detail from the perspective of those with intimate knowledge of events [22]. We conducted semi-structured interviews and focus groups with key stakeholders throughout the state of Michigan to explore institutional experiences with drug shortages, approaches to recent shortages, openness to ongoing inter-institutional engagement, ideas for a shared resource, and potential obstacles to implementation. Questions used in the interviews are shown in S1 File. This study was deemed exempt from formal review by University of Michigan’s IRBMED.

### Sample

A list of hospitals was compiled from the Michigan Health & Hospital Association (MHA), excluding long-term care facilities, government-owned institutions, psychiatric, and mental health facilities. To hear diverse experiences and ensure any collaborative resource would be valued by most hospitals, we purposefully selected 10 hospitals to form a sample with heterogeneity in the following characteristics: the number of licensed hospital beds, type of hospital (academic, community, etc.), geographic location, critical access status, trauma designation, teaching status, religious affiliation, profit classification, and larger health-system affiliation. Information about hospital characteristics was gathered from the State of Michigan (beds, critical access), the U.S. Census (rurality), the American Trauma Society and the American College of Surgeons (trauma designation), hospital websites, and other publicly available information (including religious affiliation, not-for-profit status, teaching status, larger health-system affiliation). Sample size was determined by resource constraints.

At each institution, we reached out by phone to directors of pharmacy/chief pharmacy officers and relied on them to include additional team members such as drug shortage pharmacists and/or technicians. Interviewers had no relationship to study participants prior to the study. After initial interviews, we further inquired if any additional person at their institution had a significant involvement in shortages and should be interviewed (typically ethics members). We interviewed between one and five key informants, representing (1) pharmacy; (2) clinical leadership/administration; (3) ethics; and/or (4) patient advocates or patient/family centered care representatives. Pharmacy representatives included directors, managers, clinical pharmacists, pharmacy technicians, and purchasers. Sixteen interviews included 25 participants with varying of experience managing shortages.

### Data collection

An interview guide was based on study team insights regarding concerns, approaches, and open questions, as well as review of published quantitative surveys [16, 20, 21]. The interview guide was pretested for comprehension, flow and length. Modifications were made after testing and subsequently included the following domains: (1) demographics, (2) experiences with shortages, (3) approaches to shortages, (4) interest in a statewide resource, (5) ideas for shared resources and potential obstacles, and (6) any additional information they wished to add. At least one representative from 7 out of the 10 originally identified hospitals completed an interview. Contacts for the remaining 3 hospitals did not respond to requests for an interview. After several attempts to contact, those remaining 3 hospitals were replaced with institutions similar in size, location, profit status, and other characteristics. Research staff conducted interviews at each interviewee’s workplace when possible (60%) to build rapport and encourage responses, or, when not feasible, via telephone. Six of the selected interviewees opted to include other key members of their institutions’ drug shortage management teams as additional participants in the interviews. Research staff (EC or SH) provided all participants with a brief overview of the study purpose and obtained verbal informed consent before conducting the interview. Interviews were audio-recorded and transcribed verbatim.

### Analysis

Thematic analysis of transcripts included multiple analysts and iterative coding of transcripts. Methods are reported in accordance to consolidated criteria for reporting qualitative research (COREQ) guidelines [22]. Two study team members created an initial coding scheme based on the interview guide and careful review of two interview transcripts for emerging themes. Two out of three study team members (*SH*, *IA or IM)* then independently coded each of the 16 transcripts line-by-line; discrepancies were resolved by discussion that included a third study team member (*EC)* and other study team members, to allow broad understanding of the data. Throughout, the study team iteratively revised the coding scheme and recoded transcripts as needed to ensure consistent application [23]. Finally, study team members reviewed and summarized all code excerpts in detail, with two reviewers for each code. Data were coded and tracked utilizing Dedoose 8.3.10 (SocioCultural Research Consultants, Los Angeles, CA).

### Focus groups

To consolidate and verify themes from the interviews, we shared interviewees’ ideas for shared resource(s) with three focus groups. We solicited volunteers from established meetings and conferences held by professional organizations and community groups. This provided a wide variety of practitioners working in a range of practice settings including, but not limited to, home infusion, academic medical centers, and private practices. These focus groups included: pharmacists (n = 4) from the Michigan Pharmacists Association (MPA), physicians (n = 5) from the MHA, and community leaders from the Deliberative Engagement of Communities in Decisions about Resource Spending (DECIDERS) Steering Committee (n = 5). Each focus group discussion was conducted by two study staff members (EC and SH) as well as one of two co-investigators (SK or SG). All three focus groups were audio-recorded and transcribed verbatim.

## Results

Hospital characteristics are shown in Table 1. The majority of participants were pharmacy personnel. Participants in our sample reported an average of 11 years of experience in handling drug shortages, with a range of 1 to 44 years. Among the participants interviewed from a variety of hospitals in Michigan, several themes were consistent: (1) numerous drug shortages strategies occurring simultaneously; (2) inadequate resources and lead time to proactively manage shortages; and (3) interest in, but varied attitudes toward, a collaborative approach.

**Table 1:** Hospital characteristics.

**Number of institutions**	10
	**N**
**Health-system affiliation**	
Part of a health-system	8
Not part of a health-system	2
**Hospital type**	
Academic	3
Community	7
**Geographic classification**	
Mostly Rural	1
Rural	1
Urban	8
**Critical access hospital**	
No	8
Yes	2
**Institutional religious affiliation**	
No	10
Yes	0
**Licensed beds**	
< 25	2
25–100	0
100–250	2
250–500	4
500–999	0
1000+	2
**Organization type**	
For-Profit	2
Not-for-Profit	8
**Trauma designation**	
Level I	4
Level II	3
Level III	1
Level IV	2
**Teaching status**	
No	2
Yes	8

Characteristics of qualitative interviewer’s unique institution.

### Numerous drug shortage strategies occurring simultaneously

Examples of drug shortages provided by interviewees overlapped a great deal, with the exception of select drugs that were connected to breadth of services provided. Participants expressed managing several shortages simultaneously.

Pharmacy personnel typically held recurring meetings to discuss shortages unable to be managed through a simple substitution in National Drug Code (NDC). Meeting frequency varied and ranged from daily to weekly, although impromptu meetings were scheduled occasionally for severe shortages. Eight institutions identified as belonging to a larger health-system. Overall, four unique health-systems were represented. Individuals from all of these health-systems reported that management and mitigation strategies took place at a system-wide level, while local institutions were responsible for implementation. Smaller institutions had the flexibility for frequent ad-hoc meetings, while large institutions implemented system-wide changes and thus had less frequent but more structured meetings.

Institutions with a system-wide shortage team would, at times, manage inventory by transferring drugs from hospital to hospital, while those that managed shortage decisions locally rarely engaged in such transfers. Interviewees often talked about the lack of accurate, up-to-date inventory systems, adding complexity to managing inventory at off-site locations. Ordering strategies were also common, such as backorders, to ensure supply would be shipped as soon as they were made available at the wholesaler. Another participant admitted facing significantly more shortages after the hospital was acquired and had to switch to an alternative wholesale distributor.

**Fig 1:**
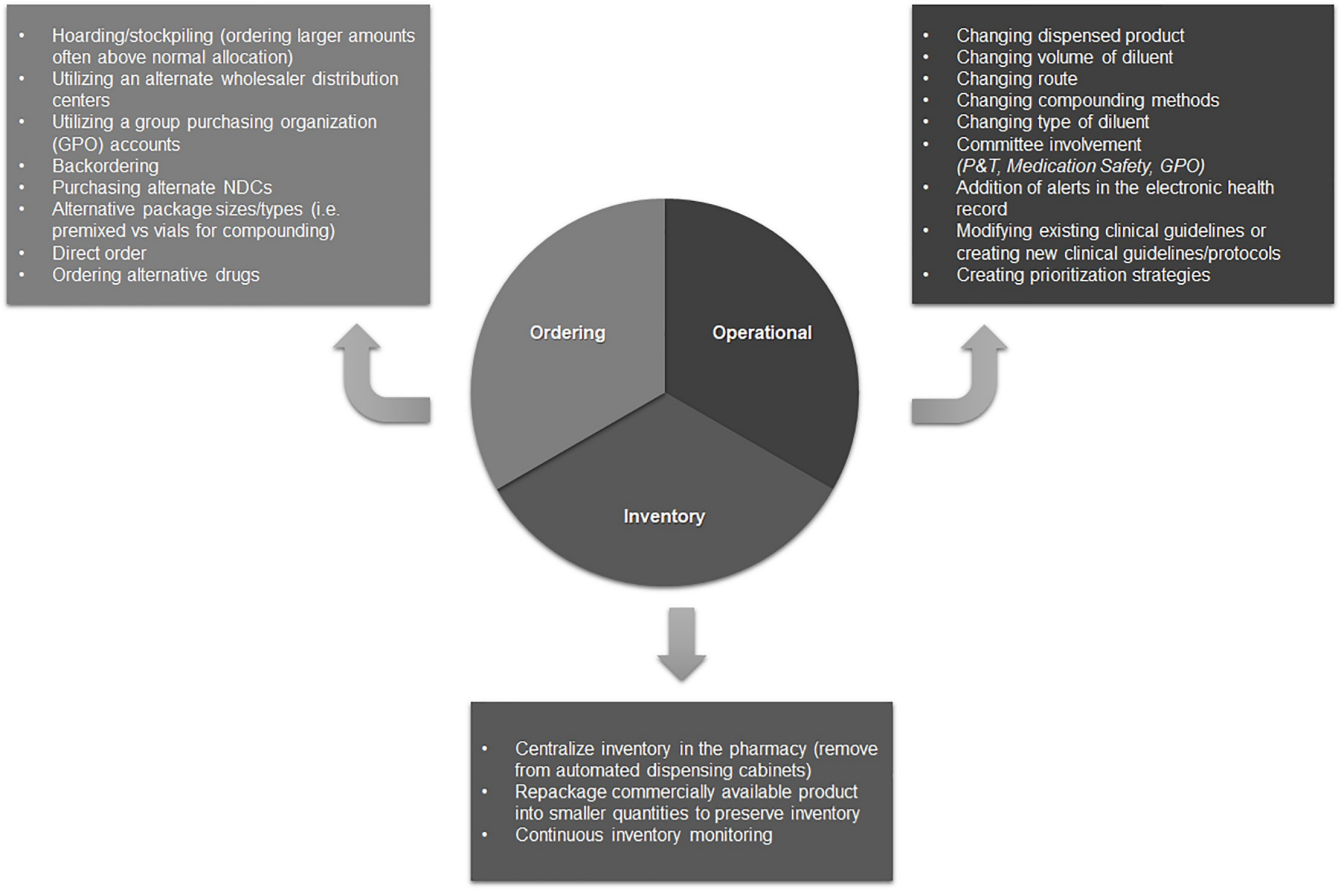
Shortage mitigation strategies. Reported hospital shortage mitigation strategies by category.

Participants reported that due to the dynamic nature of shortages, multiple strategies would be pursued, sometimes within the same day (Fig 1).

Only two institutions reported involving an ethics consultant or committee member in drug shortage planning. Patients were rarely informed about the occurrence of a drug shortage, which participants explained was due to implementing drug shortage management strategies that did not influence the patient experience or treatment. While most institutions reported shortage management rarely resulted in changes in clinical care such that patients had to be prioritized, others reported that in severe shortages, they created guidelines to prioritize patients based on acuity and drug necessity. If able, patients were switched to alternative therapies, while patients with no other therapy options either had their therapy plans extended or had to be transferred to another site to continue unchanged drug therapy plans. Participants also reported modifying the organization’s ordering and inventory strategies to limit the number of patients affected. Few institutions involved individuals with expertise in ethics to assist in determining a fair process/strategy, although participants involved in the prioritization process reported significant amounts of time dedicated towards making a fair, equitable decision.

Participants emphasized the majority of shortage management activities remained within pharmacy departments. Shortage management teams limited communication with healthcare providers and larger committees to an as-needed basis, usually only when shortages were deemed severe. Unless a shortage directly affected a patient’s treatment plan, most patients were not informed (or even aware) of ongoing shortages.

### Inadequate resources and lead time to proactively manage shortages

The majority of participants (17/25) reported obtaining information about new shortages from more than one location/resource. At several institutions, shortages were typically discovered at the time of purchase. Purchasers would communicate with pharmacy managers or shortage pharmacists regarding product availability, expected shortage duration, and alternate brands or drug preparations. Interviewees reported discovering drug shortages through distributors or manufacturers; other hospitals within the same health system (if applicable); or existing shortage management resources such as ASHP’s drug shortage list, the FDA email list, local/regional professional organization conference e-mail lists or conference calls, and group purchasing organization (GPO) e-mail lists or conference calls. Five participants who consulted existing resources expressed concerns over delays in reporting, which resulted in little to no lead time to prepare for shortages and implement mitigation strategies. Participants did acknowledge that, infrequently, they would get a notification of a shortage affecting other parts of the country that had not yet affected the State of Michigan. Largely, despite the numerous resources, shortage notifications were often delayed, out-of-date, and/or provided little information about shortage mitigation strategies.

A minority of interviewees noted that their hospital had a designated drug shortage pharmacist, but many managers, assistant directors, and directors were directly involved in the decision-making and strategy process. Institutions decided upon a shortage strategy after consideration of various alternative and substitution options. Less than half of the institutions interviewed had a pharmacist or team exclusively dedicated to researching alternative therapies. They relied on providers specializing in the shortage area to determine an appropriate therapy, resulting in changes in guidelines or procedures. If shortages were particularly severe or affected large populations, a strategy was typically created or modified, then presented to the pharmacy and therapeutics (P&T) committee. This committee then approved alternative therapeutic guidelines or drugs not on the formulary to mitigate the drug shortage.

### Interest in a collaborative resource

After describing experiences with and approaches to drug shortages, interviewees were asked to consider characteristics or features of a collaborative resource that could help manage shortages; their responses are summarized in Table 2. Overall, participants listed the critical components of an ideal resource as having a user-friendly design; involving transparency on the part of both manufacturers and institutions; encouraging and fostering broad willingness to participate; and establishing third-party ownership/control of the resource itself to assure independence from any given hospital or health system.

**Table 2:** Collaborative resource characteristics.

Characteristic	Rationale
User-friendly	Easy-to-use resource that is intuitive and specialized towards drug shortage management
Manufacturer transparency	Shortages are often noted at the time of manufacturing due to lack of raw material or sourcing issues, thus, this would provide more lead time for institutions to prepare for mitigation strategies
Institution transparency/willingness to participate	In order for a statewide resource to be successful, institutions must actively engage in conversation and participate in information-sharing
Third-party ownership/control	The resource should be owned and maintained by a third party to alleviate any potential conflicts of interest, alliance with, and bias towards institutions

When asked to describe potential barriers to the successful creation and usage of a statewide resource to share information, participants identified several common concerns, including differing levels of perceived benefit, logistical barriers to drug sharing (e.g., Drug Supply Chain Security Act [DSCSA], Drug Enforcement Agency [DEA] regulations, geographic barriers), sharing of proprietary information, ethical considerations about preferential drug distribution, resource maintenance, updating information into the resource, and interest of the hospital. Several participants echoed that the maintenance of a resource would take significant effort and admitted the lack of automation related to their inventory records would make sharing supply-on-hand difficult. Therefore, the resource would need a simple way to input information initially and continually for further updates. Other participants suggested that difficulty in selling drug(s) to other institutions in the event of a surplus, even outside of shortage situations. Additionally, hospitals may have specific product discounts by qualifying for 340B purchases, generic discounts through GPOs, or other contracts. Hospitals would not be able to sell these medications to others from a legal perspective due to the proprietary information involved. Another potential barrier to collaboration included ethical concerns regarding distribution. Even if criteria were established to ensure fair allocations, it is possible multiple hospitals could meet these criteria simultaneously, requiring the resource owners to make further difficult decisions.

On several instances, participants highlighted the competitive nature of drug shortage management by discussing the difficulty operationalizing decisions within the same health-system or noting poor inventory management strategies by other institutions such as stockpiling. If a particular hospital managed the resource, interviewees expressed concerns that that institution would be privy to a significant amount of exclusive information. Thus, many promoted the ownership of the resource by a third-party or professional organization with no direct hospital relationships.

### Focus groups

The study team compiled the list of characteristics of an ideal resource and theorized potential solutions shown in Table 3. These ideas were then presented to focus groups of pharmacists, physicians, and community members in order to generate discussion on potential facilitators and challenges involved in creating the resource. After the ideas were introduced, focus groups brought up the role of the state and federal government and the strengths and weaknesses of governmental intervention. Many suggested the government should force drug manufacturers to be more transparent about upstream drug shortage issues to reduce the downstream effect on individual institutions. The focus groups discussed drug supply chain management in other countries and suggested a system in which the government was the sole purchaser of the drugs would alleviate some drug shortage issues. They also referred to importation strategies and the need for governmental approval.

**Table 3:** Collaborative resource ideas.

Potential resources	Rationale
Physical drug repository	Institutions that have drug surplus could facilitate sharing between institutions; drug caches, which exist for statewide emergencies, could be adapted for drug shortages
Drug information database/resource	All institutions, essentially, are completing the same literature review, however, some institutions do not have dedicated personnel to do this, thus, a shared database could help expedite operationalizing strategies
Centralized inventory management	Real-time inventory records of participating institutions that would promote transparency and sharing; should include ‘supply on hand’ and ‘number of days’ supply’ to normalize inventory based on institution utilization; list could also help hospitals reduce drug surpluses
Expanded access to international products	Products may be available across international borders and could be imported for domestic use
Up-to-date shortage list	Shortage list pertinent exclusively to the state of Michigan that would provide more real-time and accurate information, including stocking dates and expected shortage durations
Online forum/discussion board	Discussion board to inquire about shortage management strategies, operational considerations, and use of alternative therapies to promote inter-institution collaboration

Pharmacists were encouraged by the potential for a regional email list that would connect drug shortage managers at each institution. Additionally, several participants indicated the benefit of increased collaboration. The group acknowledged potential challenges, doubting how transparent institutions would be with resources.

Physicians of varying specialties (e.g., pediatrics, surgery) and settings provided different insights due to their lack of involvement in daily shortage management. They acknowledged that the drugs on shortage are primarily generic drugs, an issue in the drug supply chain on a national level. When presented with potential resource ideas, the group had concerns regarding initial data entry and continued maintenance of the resource, especially when existing internal pharmacy systems require significant work to maintain.

Community members noted the lack of patient participation in times of drug shortages and were concerned about the patient voice. One participant expressed concerns about the amount of communication given to patients about drug shortages and alternative therapies. These critical times include if alternative drugs must be used or if therapies are delayed. This group expressed interest in having a resource from a patient/family perspective to locate necessary medications: “I want to know when I can get this”.

## Strengths and limitations

This qualitative assessment has several strengths and limitations. Open-ended interviews and focus groups, with informants recruited for a breadth of experience, may not reflect the “average” experience of institutions. Focus groups were led with overall discussion on shortage approaches and a list of potential resource ideas, which may have led to bias when further developing potential resource concepts. Additionally, community members were less familiar with the medication use process and drug shortage management, thus some of their discussion focused less on day to day management of drug shortages and more on external forces. Audio recording of each interview and focus group could lead to less frank answers, despite the anonymity maintained throughout the study.

Interview and focus group participants derived from selected sample hospitals resulted in a small sample size and had little representation from critical access and non-urban hospitals. This leads to difficulty extrapolating statistically significant conclusions about drug shortage experiences, approaches, and ideas about a shared resource to underrepresented institution types. Overall, the size of the hospitals in the sample based on licensed hospital beds was relative small (majority having less than 500 beds). This may limit the applicability of our conclusions to states with larger hospital bed averages. However, this is generally reflective of the hospitals in the State of Michigan. Additionally, the majority of the hospitals represented were not-for-profit. Thus, strategies regarding drug shortages discussed may overshadow opportunities afforded to only for-profit sites. Participants only highlighted participation in GPOs, thus involvement with integrated delivery networks (IDNs) were not discussed and could be a potential opportunity to investigate in the future. Data collection preceded the COVID-19 pandemic, thus recorded drug shortage mitigation strategies may not be wholly consonant with current experience.

Open-ended data collection, however, also brings strengths. In interviews, the participants were extremely knowledgeable with vast experience. The focus groups benefitted from relaxed group dynamics and enabled an assessment of themes and ideas found in interviews from different perspectives. Lastly, while some participants (e.g., community members) may not have been very involved in drug shortage management, their comments regarding the concepts revealed larger issues about external communication and education.

## Discussion

Our sample described similar experiences with shortages and themes related to shortage management [24]. Inaccuracies and lag-time of the current shortage lists suggest the need for real-time shortage information to be communicated to hospitals. Additionally, shortages tend to affect regions based on supply chain constraints, forcing clinicians to react to shortages in lieu of proactively managing drug shortages or inventory. Frequently, personal or professional networks help provide insight, and, less often, even product. Due to the legal complexities of drug borrowing/distribution, borrowing is often restricted to urgent/dire situations and never resolves the shortage. Standalone institutions, not part of a large GPO, and institutional leaders not involved in professional organizations, seem to have less opportunity for collaboration. This predicament highly suggests the need for a collaborative resource that is established on a regional or state-wide level.

ASHP Drug Shortage Guidelines outlines a standard of process on drug shortage management, however, the actual management inevitably varies due to lack of resources needed to investigate, brainstorm, and monitor drug shortages. Thus, as drug shortage management often happens in concert across institutions, it would be beneficial for institutions to share not only information about drugs facing shortages, but also information regarding substitutions and clinical alternatives. Some national resources attempt to create online member-only forums, which tend to also include non-drug-related shortages and other clinical problems. However, these member-only opportunities isolate non-members and prevent full statewide/regional collaboration. Any future resources created should be open to all drug shortage managers at institutions in a designated area, however, should be somewhat restricted to prevent non-drug shortage-related matters or membership should be monitored closely.

Despite enthusiasm of participants to engage in a shared resource, many expressed concerns regarding variance in participation based upon institutional size/resources, and hesitancy to divulge sensitive information as potential barriers. It is recommended that future resources are maintained by an external third-party or by individuals that are not affiliated with institutions facing shortages. Additionally, a different resource for patients, families and communities is also needed, with care given to make it accessible for all. Such a resource should consider avoiding a “first come, first served” model toward the allocation of scarce drugs that would exacerbate health inequities.

The scope and consequences of drug shortages are vast and highlight the critical need for the community to put theory into practice by establishing systems to ensure equitable and just treatment of affected individuals [25]. Institutional practices, even if well-informed by ethical theory, need empirical research examining impact to see if just distribution of scarce resources is enhanced or undermined and whether decision making about scarce resources meets standards of procedural justice.

Any collaborative tool to be used will only remedy the complications of drug shortage management and will not bring more drug into circulation. Thus, it would be careless to dismiss the need for intervention on a larger level. Health care professionals should continue to promote the transparency from wholesale distributors and pharmaceutical manufacturers, in order to deliver care to patients reliably.

## Conclusions

The breadth and impact of drug shortages pose a formidable challenge to providing consistent, quality medical care. The qualitative interviews provided profound insight that will be utilized to help develop, implement, and test a real-world, practical tool for enhancing the fair allocation of scarce drugs. This study highlights necessity and opportunity for inter-institution collaboration through a shared resource, which may help institutions adopt accepted best practices. A collaborative resource may also more efficiently determine the state of supplies in regional locales or statewide to determine how best to access or share finite resources in times of shortage. Promoting cooperation with drug shortages is also likely to pave the way for further knowledge sharing in various aspects relevant to patient care.

## Supporting information

S1 File Qualitative interview guide. Script followed when conducting qualitative interviews.
(PDF)

S2 File COREQ checklist. Original journal article describing COREQ checklist.
(PDF)
