# Supplementary material for: Drug shortage management: A qualitative assessment of a collaborative approach
Source: PLoS One. 2021 Apr 23;16(4):e0243870. doi: 10.1371/journal.pone.0243870 (PMC8064571; doi:10.1371/journal.pone.0243870)
Supplement: S1 File — Script followed when conducting qualitative interviews. (PDF) [file pone.0243870.s001.pdf]

Statewide Approaches to Drug Shortages

Interview Script

Thank you so much for agreeing to speak with me today. My name is _________ and I am part of a research team at the University of Michigan.

As you know we are talking to people working in and with hospitals in Michigan to learn more about the problem of drug shortages and how hospitals are handling these shortages. As you are aware, drug shortages can be frequent and major barriers to providing consistent and high quality medical care. We hope to identify and describe best practices and propose a possible statewide resource to mitigate some of the challenges associated with shortages.

This interview will take about 30 – 45 minutes and includes questions about your experience with drug shortages, how they are handled, and thoughts about a statewide resource. Please know that your participation is voluntary and you can discontinue participation at any time or refuse to respond to specific questions. We will record each interview. We will not use your name or other information that could identify you. This research project is funded by the Blue Cross Blue Shield of Michigan Foundation.

Do you have any questions? *[If none]* Do you agree to participate? *[If yes, then proceed with recording.]*

Section 1: Demographics & Current Position

1. If you are comfortable sharing general demographics, would you be able to provide your gender, age, and race/ethnicity?
2. Tell me about your professional field/training and current position.
3. How many years have you been involved in drug shortages, including any previous positions or training?

Section 2: Experiences with Drug Shortages

4. What is your role and experience in addressing drug shortages?
5. How many years have you been involved in managing drug shortages?
6. Could you tell me about a drug shortage you remember that occurred while in your current role?
7. Two shortages which have occurred in the past year involve mini-bags of intravenous fluid and etoposide. Can you tell me whether and how both of these affected your institution, if at all?

Section 3: Approach to Shortages

8. Who—what person or group—at your institution is responsible for addressing drug shortages on a day-to-day basis?
    a. [If group] How many are involved?
    b. Who is the first-line responder?
        i. First person to *hear* about the shortage?
        ii. First person to *act* on the shortage?
    c. What kind of training does this person/group have?
    d. How much time does this person/group spend?
    e. How are others integrated?

9. **Other than those involved in day-to-day shortage management, who else gets involved when a shortage occurs?**
    a. Do you have a dedicated person, how many, what level of training?
    b. How much time do they spend?
10. **Does your institution have an established policy or procedure that deals with drug shortages?**
    a. What does it cover?
        i. Would it be possible to receive a copy of that policy/procedure?
    b. Can you summarize what it says?
    c. How do you think it's working?
        i. What are the strengths of this policy?
        ii. What are the weaknesses?
11. **[for system hospitals] How are drug shortages managed locally at this institution compared to system-wide?**
12. *{Earlier you mentioned that your institution sometimes identifies substitutes or alternatives to use in place of the drug in shortage.}* **Could you talk [more] about whether and how alternatives/substitutes are considered/chosen?**
    a. **Could you tell me about any times when this did not happen?**
13. **Outside of the Pharmacy Department, what resources help your institution address the issue of drug shortages? [probes: internal, external, system]**
14. **Earlier you mentioned that your institution has had to prioritize patients' access to scarce drugs.**
    a. What do you think about those decisions and how they are working? *[probe for examples]*
    b. How is this information communicated to providers? To patients? *[probe for examples]*
    c. What is your approach to inventory management?
        i. How are inventory levels adjusted?
            1. Prior to a shortage?
            2. During a shortage?
            3. After a shortage?
15. **What have been the financial implications of drug shortages?**
    a. What are the estimated costs of managing drug shortages? *[probe about FTE, drug pricing]*
16. **How is drug shortage information disseminated to providers? To patients?**

Section 4: Interest in a Statewide Resource

17. **Do you currently communicate with anyone outside your institution in response to drug shortages?**

a. If so, how? With whom? Through what means? How often? *[probe for details]*
b. If not, is this something you would like to be able to do? For what reason(s)?

18. How do you think we could improve communication and collaboration across healthcare systems?
    a. What level of resource would be most helpful? *[local, regional, state, national]*
19. Do you think a statewide resource to improve communication and collaboration would help you and your institution address drug shortages?
    a. If so, what do think it could help with? What would you like to see it do?
    b. If not, why not? *[probe – for example, competition might come up, so ask how that could influence the use of the resource and how that obstacles might be overcome]*
20. What ideas do you have for this kind of resource? What would make it welcome and easy to use for you and others at your institution?
    a. Probe about website, regular meetings, email list-serve, etc.
21. What barriers or facilitators do you foresee, if any, in being able to develop such a resource?
22. What barriers or facilitators do you foresee, if any, in being able to use such a resource?

Section 5: Concluding Remarks

23. Do you have any additional questions or comments?
24. Is there anyone else at your institution you think we should speak with?
25. Can you provide contact information for individuals you know at other institutions who might want to participate?
26. Can we contact you again with additional questions or follow-up?
